# β-carboline biomediators induce reveromycin production in *Streptomyces* sp. SN-593

**DOI:** 10.1038/s41598-019-42268-w

**Published:** 2019-04-09

**Authors:** Suresh Panthee, Shunji Takahashi, Teruo Hayashi, Takeshi Shimizu, Hiroyuki Osada

**Affiliations:** 1 RIKEN Center for Sustainable Resource Science, Natural Product Biosynthesis Research Unit, Wako, Hirosawa 2-1, 351-0198 Saitama, Japan; 2 RIKEN Center for Sustainable Resource Science, Chemical Biology Research Group, Wako, Hirosawa 2-1, 351-0198 Saitama, Japan; 3 Present Address: Teikyo University Institute of Medical Mycology, Otsuka 359, Hachioji, Tokyo Japan

## Abstract

The biosynthetic potential of soil-dwelling actinomycetes to produce diverse bioactive molecules that are useful as drug seeds has been achieved in the laboratory by modifying culture conditions. Availability of a small molecule that can induce secondary metabolism in these microbes can greatly facilitate the exploration of bioactive natural products. In this manuscript, through the screening of natural products and chemical modification, we demonstrated that the presence of the β-carboline compound, BR-1, enhanced reveromycin A production in *Streptomyces* sp. SN-593. BR-1 induced reveromycins production at the wide range of concentrations without affecting cell growth. Our study indicates that BR-1 might serve as an alternative to activate specialized metabolite biosynthesis without genetic engineering.

## Introduction

Soil dwelling actinomycetes are characterized by their ability to produce diverse specialized metabolites (SMs), that account for majority of drugs presently in clinical use^1^. These bacteria utilize autoregulators such as A-factor^2^, 2-alkyl-4-hydroxymethylfuran-3-carboxylic acids^3^, PI factor^4^, avenolide^5^, L-factor^6^, VB-A^7^, IM-2^8^, SCB1^9^, and SRB1^10^ to induce morphogenesis and regulate SM production. As opposed to autoregulators, chemical signals derived from extra-species/environmental stimuli such as hormaomycin^11^, goadsporin^12^, promomycin^13^, antibiotic-remodelling compounds (ARCs)^14^, and rare earth elements such as scandium^15^ can also induce morphogenesis and SM production in *Streptomyces* species. Activation of SM gene clusters by small molecule elicitors has also been observed for Gram-negative proteobacteria *Burkholderia thailandensis*^16^.

Classically, induction of SM production in *Streptomyces* has been achieved by modifying culture medium and recently ribosomal engineering^17,18^, expression of key regulatory genes^19,20^, and heterologous expression^21^ have also been used for the same. By using the classical approach of culture medium modification, we found that reveromycin (RM) production by *Streptomyces* sp. SN-593 was enhanced by adding tomato juice to the culture medium^22^. This observation led us to speculate that naturally existing extracellular chemicals can activate secondary metabolism. Such chemicals can up-regulate SM biosynthesis and facilitate the isolation of novel natural products without genetic engineering. We first tried to purify the active principle from tomato juice. However, all attempts failed regardless of extended efforts. A major obstacle impeding the identification of natural chemical signals is their low-level presence in natural environments. Therefore, screening a natural product library is an alternative approach for finding core chemical structure that enhances SM production. Given that RM-A has antifungal activities against pathogenic plant fungi^23^ and unique biological activity inducing the morphological reversion of *src*^ts^-NRK cells from spherical transformed cells to flat normal cells^24^, we used both assay systems to screen for compounds that enhance the production of RMs. By the screening of small molecules from the RIKEN Natural Products Depository (NPDepo)^25^, we identified a β-carboline lead compound that enhanced RM production. Based on structure activity relationship study, we succeeded to create novel chemical signals that activate RM production at sub μM concentration.

## Results and Discussion

### Screening for biomediators

**Figure 1:**
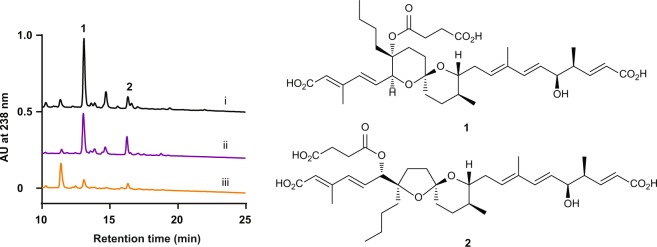
LC-MS analysis of RMs after treatment with NPD2639. *Streptomyces* sp. SN-593 was analysed after treatment with 5% tomato juice (i), 1 µg ml^−1^ NPD2639 (ii), or 0.1% DMSO (iii).

We defined extracellular chemical signals as biomediators to distinguish them from autoregulators. After the failed attempt of purification from tomato juice, we utilized the antifungal activity of RM-A to screen overproduced RMs after the treatment of NPDepo compounds. The activity of putative hits was further analysed by the *src*^ts^-NRK cells assay followed by liquid chromatography-mass spectrometry (LC-MS) analysis to quantify the production of RM-A (**1**) and its derivative RM-B (**2**). RIKEN NPDepo harbours approximately 26,000 compounds. Of this, 3,155 compounds were screened using an antifungal assay with *Pyricularia oryzae*, the hits (136 compounds) were further examined in the *src*^ts^-NRK cell morphological assay. We also performed liquid chromatography-mass spectrometry (LC-MS) analysis to quantify the production of RM-A (**1**) and its derivative RM-B (**2**). Finally, we succeeded in identifying a β-carboline compound NPD2639 (**3**) as a lead compound (Fig. 1).

### Structural insights into the biomediator activity of β-carbolines

**Figure 2:**
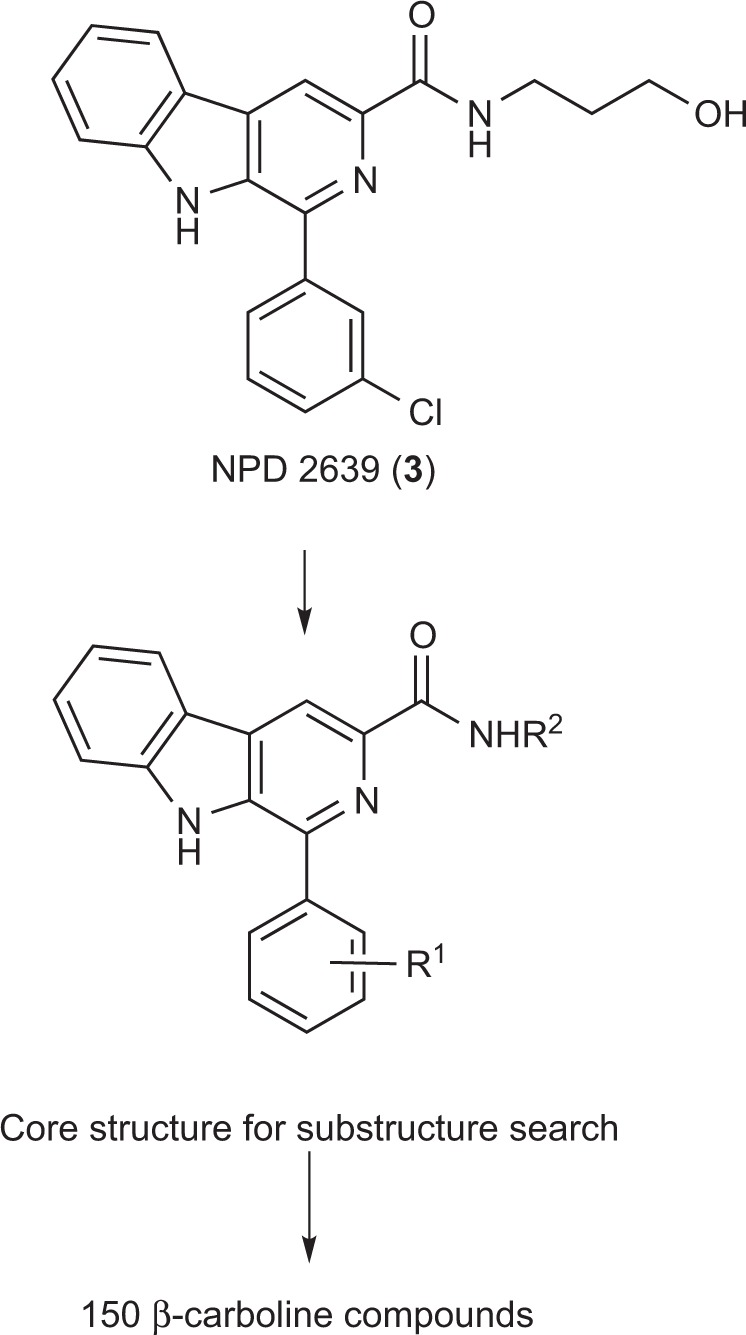
Substructure search for similar compounds in an NPDepo chemical library.

To elucidate the structure activity relationship of the biomediator, we first investigated the biomediator activity of the β-carboline core structure using natural products such as harman, harmol, and harmine. These compounds did not induce the production of RMs (Table 1). This result suggested a functional role for the 3-chlorophenyl group attached to 1 position and the amide group attached to the 3 position of the β-carboline core, in terms of biomediator activity. To investigate the structure–activity relationship, we selected 150 β-carboline compounds from the RIKEN NPDepo library^25^ with regard to the substituents at the 1-phenyl group (R^1^) and the 3-amide moiety (R^2^) (Fig. 2). The biomediator activity was evaluated in the *src*^ts^-NRK cells assay followed by LC-MS analysis of RM production (Table 2). The hydroxyethyl derivative (**4**) and 3-bromophenyl derivatives (**8s** and **8t**) also retained biomediator activity. However, the 2 or 4-substituted phenyl derivative at R^1^ and compounds with substituents other than hydroxyalkylcarboxamide at R^2^ did not have strong biomediator activity. Moreover, the hydroxyethyl carboxamide derivatives at R^2^ (**4** and **8t**) had better biomediator activity compared to their hydroxypropyl carboxamides (**3** and **8s**) (Table 2). Based on these results, we speculated that the chain length of the hydroxyalkyl group attached to the carboxamide moiety was key for biomediator activity.

**Table 1:** Natural β-carboline compounds and their biomediator activity. RMs produced after the treatment of 1 μg ml^−1^ compounds was quantified by LC analysis. The data shown are expressed as the mean ± SD from 3 experiments.


R	RMs mg l^−1^
H (harman)	12 ± 7
OH (harmol)	19 ± 5
OMe (harmine)	14 ± 3

**Table 2:** Structure–activity relationships of β-carboline compounds.

		R^2^											
											
R^1^		(+)	(+)							(−)	(+)		
			(−)						(+)				
			(−)										
		(−)	(−)	(+)	(−)		(−)	(−)	(−)			(−)	
		(−)		(+)				(+)	(+)	(+)			
		(+)											(−)
		(+++) NPD2683 (**4**)	(++) NPD2639 (**3**)			(−)		(−)		(++)	(−)		(−)
		(+++) (**8t**)	(++) (**8s**)		(+)		(+)	(+)	(+)	(+)		(−)	
		(−)					(+)				(+)	(+)	

A *src*^ts^-NRK cell assay and HPLC analysis were performed to evaluate the RM production after the treatment with 1 μg ml^−1^ compounds from substructure search. The biomediator activity of 41 out of 150 β-carbolines is summarized, enabling a focus on the key structures involved. (−), no activity in *src*^ts^-NRK cell assays, and RM production was not measured by HPLC; (+), positive in *src*^ts^-NRK cell assays, and RM production was less than 40 mg l^−1^; (++), positive in *src*^ts^-NRK cell assays, and RM production was 40–50 mg l^−1^; (+++), positive in *src*^ts^-NRK cell assays, and RM production exceeded 50 mg l^−1^.

### β-carboline structure optimization

To optimize the structure of seed compound **3** for inducing RM production, we synthesized derivatives with regard to the amide group and the aromatic substituents (Table 3–5, Fig. S1). First, while retaining the 3-chlorophenyl group at R^1^, we modified the R^2^ group to afford **8a–8d**. The acetate (**8c**) and methyl ether (**8d**) groups did not exhibit biomediator activity. Whereas the *N*-hydroxycarboxamide (**8b**) retained biomediator activity, the maximum yield of RMs (72 ± 12 mg/l) was obtained with the simple carboxamide (**8a**).

**Table 3:** Structure–activity relationships of β-carboline compounds that enhanced the production of RMs: Modifications of the substituents attached to the phenyl group or pyridine ring. *Streptomyces* sp. SN-593 was cultured separately in the presence of 1 µg ml^−1^ of each compound. RM production was measured by LC analysis. The data shown are expressed as the mean ± SD from 3 experiments.


Compound	R^1^	R^2^	RMs mg l^−1^
**3** (NPD2639)	3-Cl	CONH(CH_2_)_3_OH	50 ± 6
**4** (NPD2683)	3-Cl	CONH(CH_2_)_2_OH	61 ± 10
**8a**	3-Cl	CONH_2_	72 ± 12
**8b**	3-Cl	CONHOH	57 ± 7
**8c**	3-Cl	CONH(CH_2_)_3_OAc	16 ± 6
**8d**	3-Cl	CONH(CH_2_)_3_OMe	2 ± 2
**8e** (BR-1)	H	CONH_2_	84 ± 5
**8f**	3-OMe	CONH_2_	57 ± 4
**8g**	3-F	CONH_2_	68 ± 1
**8h**	3-Br	CONH_2_	63 ± 4
**8i**	3-NO_2_	CONH_2_	22 ± 3
**8j**	4-Cl	CONH_2_	25 ± 5
**8k**	4-Cl	CONHOH	18 ± 9
**8l**	3,5-diF	CONH_2_	37 ± 4
**8m**	3,5-diCl	CONH_2_	22 ± 6
**8n**	3,5-diBr	CONH_2_	18 ± 6
**8o**	H	H	2 ± 1
**8p**	H	CONH(CH_2_)_3_OH	39 ± 15
**8q**	H	CONH(CH_2_)_2_OH	22 ± 6
**8r**	H	COOH	0.85 ± 0.23
**7a**	H	COOMe	0.35 ± 0
**8s**	3-Br	CONH(CH_2_)_3_OH	48 ± 26
**8t**	3-Br	CONH(CH_2_)_2_OH	60 ± 23

**Table 4:** Structure–activity relationships of β-carboline compounds that enhanced the production of RMs: Replacement of the phenyl group with other substituents. *Streptomyces* sp. SN-593 was cultured separately in the presence of 1 µg ml^−1^ of each compound. RM production was measured by LC analysis. The data shown are expressed as the mean ± SD from 3 experiments.


Compound	R^3^	RMs mg l^−1^
**11a**		32 ± 12
**11b**		9 ± 5
**11c**		18 ± 11

**Table 5:** Structure–activity relationships of β-carboline compounds that enhanced the production of RMs: Modification of tetrahydro β-carboline compounds. *Streptomyces* sp. SN-593 was cultured separately in the presence of 1 µg ml^−1^ of each compound. RM production was measured by LC analysis. The data shown are expressed as the mean ± SD from 3 experiments.


Compound	R^1^	R^2^	R^3^	RMs mg l^−1^
**12a**	H	COOH	H	0.69 ± 0.59
**12b**	H	CONH_2_	H	2.16 ± 1.24
**12c**	H	COOH	CH_2_OH	4.54 ± 1.82
**12d**	H	CONH_2_	CH_2_OH	4.16 ± 0.25
**12e**		CONH_2_	H	4.52 ± 1.27
**12f**		COOH	H	2.41 ± 0.18

To examine the activity of substituents in the phenyl ring, we next modified the 3-chlorophenyl group of **8a** with various aromatic rings, including 3-H (**8e**), 3-OMe (**8f**), 3-F (**8g**), 3-Br (**8h**), and 3-NO_2_ (**8i**). Except for **8i**, the other compounds (**8e–h**) retained biomediator activity, thereby highlighting the importance of substituents at position 3. In agreement, when we replaced the 3-chlorophenyl group of **8a** and **8b** with the 4-chlorophenyl group present in **8j** and **8k**, the biomediator activity decreased. Because 3-substituted phenyl derivatives retained activity, we expected activity with the 3,5-diflurophenyl (**8l**), 3,5-dichlorophenyl (**8m**), and 3,5-dibromophenyl (**8n**) derivatives. However, we found that all these derivatives had reduced biomediator activity. To examine the role of the carboxamide moiety of **8e**, we replaced it with hydrogen (**8o**), hydroxypropyl carboxamide (**8p**), hydroxyethyl carboxamide (**8q**), carboxylic acid (**8r**), or carboxylate (**7a**) moieties. The biomediator activities of **8p** and **8q** were partially retained, but **8o**, **8r**, and **7a** showed abolished activity, revealing the importance of the carboxamide moiety for the activity (Table 3). In addition, we replaced the phenyl group with cyclohexane (**11a**), furan (**11b**), or pyridine (**11c**) and found that these groups abolished the biomediator activity (Table 4). Then, to evaluate the effect of the β-carboline core, we also synthesized tetrahydro β-carboline compounds (**12a–f**). Interestingly, they had abolished biomediator activity (Table 5). Based on this structure–activity-relationship study, **8e**, named BR-1 (Biomediator that induce Reveromycin), was the most potent biomediator.

### Biological activity of BR-1

**Figure 3:**
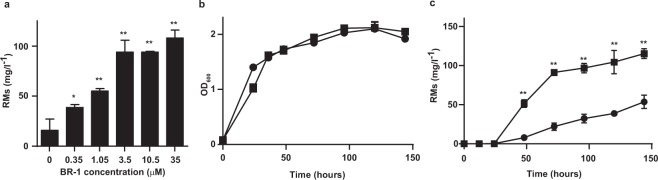
Effect of BR-1 on RM production and cell growth in *Streptomyces* sp. SN-593. (**a**) Dose-dependent RM production by BR-1. (**b**) Cell growth in SY medium (•: DMSO, ▪: 3.5 µM BR-1). (**c**) Time-dependent RM production after BR-1 treatment (•: DMSO, ▪: 3.5 µM BR-1). RM production was quantified by HPLC (data expressed as the mean ± SD from 3 experiments). *p < 0.05; **p < 0.0001 by 2-way analysis of variance.

We examined the dose dependent and time dependent biomediator activity of BR-1. The lowest concentration of BR-1 that induced RM production was 0.1 µg/ml (0.35 μM), and it produced RMs over a wide range of concentrations (0.35 μM–35 μM) (Fig. 3a). There is a general notion that a reduction in growth rate, if not growth cessation, is an important signal for triggering secondary metabolism^26^. We found that cell growth of *Streptomyces* sp. SN-593 was not affected by treatment with BR-1, suggesting that the induction of RM production was neither due to an increased cell density nor due to a reduced growth rate (Fig. 3b). Moreover, BR-1 induced RM production from day 2 and continued through day 6, compared to non-treated samples (Fig. 3c).

The RM biosynthetic gene cluster in *Streptomyces* sp. SN-593 harbors 21 genes, including the genes involved in biosynthesis of polyketide core structure, post-PKS modification, and transcriptional regulators. Among the regulators, RevQ and RevU belong to the *Streptomyces* antibiotic regulatory protein and LuxR family regulators, respectively. These two families of regulators are well known to positively control secondary metabolites biosynthesis^26^. Previously, we have shown that the constitutive expression of *revQ* gene by *aphII* promoter resulted in about 5-fold increase in the amount of RMs produced^27^. While the *Streptomyces* sp. SN-593 produces RMs at basal level in SY-B medium (Figs 1, 3a,c), the presence of 3.5 µM BR-1 enhanced RMs production about 6-fold at day 3. The fold increment of RMs production after BR-1 treatment was almost similar to that of transformed cells which constitutively expressed transcriptional regulators^27^. Based on the similar observation, we speculated that BR-1 response goes through the pathway specific regulator associated with the RM biosynthetic gene cluster.

In summary, we identified β-carboline compounds as the biomediators of RMs production. Considering that β-carboline alkaloids are widely distributed in the environment^28–30^, our study highlights the possible presence of biomediator-microbe communication in nature and these chemicals may trigger a variety of responses, including the production of bioactive molecules in microbial communities. Furthermore, our study indicates that these signals might serve as an excellent approach to activate SM biosynthesis without genetic engineering. In future, SM production by biomediator and its application to approaches such as single cell multiplexed activity metabolomics might link with the discovery of effector molecules to human cells^31^.

## Materials and Methods

### Chemical library

A chemical library from the RIKEN Natural Products Depository (NPDepo) (http://www.npd.riken.jp) was used.

### Culture medium and biomediator treatment

*Streptomyces* sp. SN-593 was cultured using several media, including synthetic medium^21^, SK2^22^, MS^22^, RM-PM^22^, SY^22^, and SY-B medium (1% soluble starch and 0.1% yeast extract). *Pyricularia oryzae* Kita1 was cultured in oatmeal agar (7.25% oatmeal agar), YG medium (2% glucose and 0.5% yeast extract), and PD medium (2.4% potato dextrose broth and 0.2% agar).

Wild-type *Streptomyces* sp. SN-593^23^ was used to screen for biomediators. Spores were prepared on MS plates. A loopful of *Streptomyces* sp. SN-593 spores was grown in 70 ml SK2 medium in a 500-ml cylindrical flask at 28 °C at 150 rpm to an OD_600_ of 6–8, 1 ml of which was diluted in 100 ml SY-B medium. A 1-ml aliquot of this mixture was prepared on a 2.2-ml well of a 96-well plate (4titude, reorder # 4ti-0130). One microliter of each NPDepo compound, dissolved in dimethyl sulphoxide (DMSO) at 1 mg ml^−1^, was added per well. Cells were cultured at 28 °C at 1000 rpm (TAITEC, BioShaker M-BR-024). After 3 days, 0.5 ml of acetone was added to each SY-B culture and centrifuged at 5,000 × *g* for 10 min (Allegra® X-15R, Beckman Coulter). The supernatant (200 µl, acetone fraction) was dried and dissolved in water (20 µl) to prepare biomediator-treated broth (BTB). Enhanced RM production was evaluated with *P. oryzae* and *src*^ts^-NRK cells assay. To quantify RM production, the acetone fraction was analysed by LC-MS. Because of non-enzymatic RM-A conversion (**1**) into RM-B (**2**), which is a 5,6-spiroacetal derivative of **1**^22^, both **1** and **2** were quantified by LC-MS analysis.

### *P. oryzae* screening system

Two agar blocks (~1 mm^2^) of *P. oryzae* Kita1 grown on oatmeal agar plates were mixed with 10 ml YG medium in a 50-ml Falcon tube, vortexed 2 min, and incubated at 27 °C at 150 rpm. After 3 days, the culture was vortexed for 2 min, diluted 50-fold in PD medium, and 200-µl aliquots were added in each well of a 96-well plate. Because RM-A inhibited *P. oryzae* growth at 5 µg ml^−1^, the optimum amount of BTB was added to each well, and the cells were cultured at 28 °C for 2 days to study growth inhibition.

### *src*^ts^-NRK cells assay system

RMs induce the morphological reversion of *src*^ts^-NRK cells from spherical transformed cells to flat normal cells when cultured at 32 °C in Eagle’s minimal essential medium supplemented with 10% calf serum. RM-A, RM-C, and RM-D exhibited EC_5O_ values of ~1.58 µg ml^−1^ for the reversal^32^. Cell aliquots (1.6 × 10^4^/200 µl) were seeded in separate wells of a 96-well plate and incubated at 32 °C for 5 h. Five microliters of BTB was added to each well, and the cells were incubated for 2 days. Then, phenotypic changes of cells were assessed microscopically.

### LC-MS analysis

Analysis of metabolites was performed by ESI-MS analysis using a Waters Alliance high-performance liquid chromatography (HPLC) system equipped with a mass spectrometer (Q-Trap; Applied Biosystems)^22^. The HPLC system consisted of an XTerra^®^MSC18 (5-μm, 2.1 mm internal diameter × 150 mm length) column maintained at 0.2 ml min^−1^. Solvent A was 0.05% aqueous formic acid and solvent B was acetonitrile. The sample was injected into the column after pre-equilibration with 30% solvent B; the column was developed with a linear gradient from 30% to 100% solvent B over 20 min and maintained in 100% solvent B for 20 min. Mass spectra were collected in ESI-negative mode.

### Chemical synthesis of β-carboline derivatives

First, β-carboline derivatives **8a–d** related to the 3–substituent were designed and synthesized^33^ from L-tryptophan and 3-chlorobenzaldehyde. Synthetic route (Fig. S1) involved the key step of the Pictet–Spengler reaction followed by oxidation with trichloroisocyanuric acid to give methyl β-carboline 3-carboxylate **7d**. The 3-carboxylate **7d** was then treated with various amines to give the desired β-carboline 3-carboxamides (**3**, **4**, and **8a**, **b**). The hydroxypropyl derivative of **3** was converted to acetate **8c** and methyl ether **8d**. Because carboxamide **8a** derived from ammonium hydroxide was the most active compound among **3**, **4**, and **8a–d**, a series of methyl β-carboline 3-carboxylates (**7a**–**j**) were synthesized and converted to the corresponding carboxamides **8e–j** and **8l**–**n**, having an amide group derived from ammonium hydroxide. The chemical structures of all β-carboline derivatives were confirmed by ^1^H NMR and high-resolution-MS data (Supporting Text).

## Supplementary information

Supporting information

## Data Availability

The data that support the finding of this study are available from the corresponding authors upon request.
