# Supplementary material for: β-carboline biomediators induce reveromycin production in Streptomyces sp. SN-593
Source: Sci Rep. 2019 Apr 9;9:5802. doi: 10.1038/s41598-019-42268-w (PMC6456619; doi:10.1038/s41598-019-42268-w)
Supplement: Supplementary file 1 — Supporting information [file 41598_2019_42268_MOESM1_ESM.pdf]

Supporting Information to:

β-carboline biomediators induce reveromycin production in *Streptomyces* sp. SN-593

Suresh Panthee[1], Shunji Takahashi[1,*], Teruo Hayashi[2], Takeshi Shimizu[2], and Hiroyuki Osada[2,*]

Affiliations

[1] RIKEN Center for Sustainable Resource Science, Natural Product Biosynthesis Research Unit, Wako, Hirosawa 2-1, 351-0198 Saitama, Japan

[2] RIKEN Center for Sustainable Resource Science, Chemical Biology Research Group, Wako, Hirosawa 2-1, 351-0198 Saitama, Japan

* Correspondence: Tel: +81-48-467-9541; Fax: +81-48-462-4669; Email: shunjitaka@riken.jp; hisyo@riken.jp

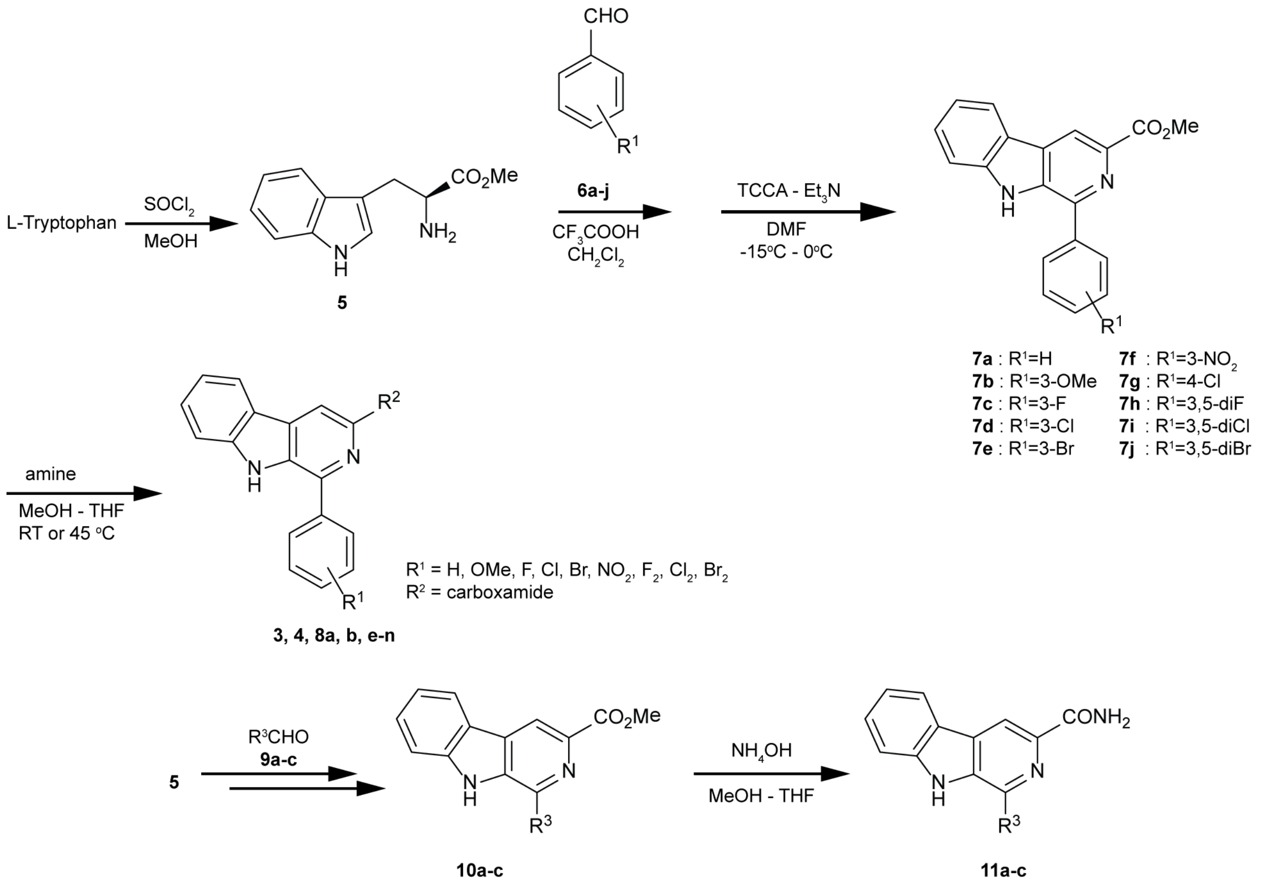

Figure S1. Synthesis scheme for producing β-carboline derivatives 3, 4, 8a, b, e – n, and 11a – c

Supporting Text

Synthesis of β-carboline derivatives.

All commercially available chemicals were used for chemical synthesis without further purification. All reactions were monitored by thin-layer chromatography (TLC) with 0.25-mm, pre-coated silica gel plates ($60F_{254}$ Art 7515; Merck, Darmstadt, Germany). For preparative layer chromatography (PLC), $60F_{254}$ Art 5744 (0.5 mm) and $60F_{254}$ 13895 (1 mm) plates were used, and for column chromatography, silica gel 60N (Kanto Chemical Co., Inc., Tokyo, Japan), NH-Silica gel (Fuji Silysia Chemical, Ltd., Aichi, Japan) and Cosmosil 140C18-OPN (Nacalai Tesque Inc., Kyoto, Japan) columns were utilized. ^{1}H NMR spectra were recorded on JEOL JNM AL-300 and 400, and ECA-500 spectrometers. The following abbreviations are used to explain the multiplicities: s, single; d, doublet; t, triplet; q, quartet; m, multiplet; br, broad. Fast-atom bombardment (FAB) mass spectra were obtained using a JMS-HX 110 mass spectrometer. Electrospray ionization (ESI) mass spectra were obtained on a BioApex II Fourier transform ion resonance cyclotron mass spectrometer (Brucker Daltonics). Electron ionization (EI) mass spectra were obtained using a JMX-SX102A mass spectrometer (JEOL).

Synthesis of carboxylates 7a – j

General procedure. A solution of aldehyde **6** (1 mmol) in dry CH_2Cl_2 (1 ml) was added to a stirred suspension of L-tryptophan methyl ester **5** (1 mmol), CF_3COOH (0.02 ml), and well-dried molecular sieves (4 Å, 400 mg) in dry CH_2Cl_2 (10 ml) under N_2 atmosphere, and stirred overnight. After the whole mixture was filtered and washed with CH_2Cl_2, the combined organic solution was evaporated. The residue was taken up with *N,N*-dimethylformamide (DMF) (2 ml) and neutralized with triethylamine (TEA). The tetrahydro-β-carboline intermediate was subjected to further oxidation without purification. After TEA (0.5 ml) was

added, the solution was cooled to –15ºC, and a solution of trichloroisocyanuric acid (1 mmol) in DMF (1 ml) was added slowly. After the reaction mixture was stirred for 15 min at –15ºC, the reaction temperature was gradually warmed to 0ºC (1.5 h) and stirred for an additional 1.5 h at 0ºC. The solvent was removed under reduced pressure, and ice water (25 ml) was poured with vigorous stirring to give precipitated compound **7**. Compound **7** was further purified by recrystallization (methanol [MeOH] or MeOH/CH_2Cl_2) or PLC.

Methyl 1-phenyl-9H-pyrido[3,4-b]indole-3-carboxylate (7a). A colourless solid. 52%. ^{1}H NMR (400 MHz, d_6-DMSO) δ 3.93 (s, 3H), 7.33 (dd, J = 7.6, 6.8 Hz, 1H), 7.54 – 7.70 (m, 3H), 7.63 (d, J = 7.6 Hz, 2H), 8.02 (d, J = 7.6 Hz, 2H), 8.43 (d, J = 8.0 Hz, 1H), 8.93 (s, 1H), 11.94 (s, 1H)

Methyl 1-(3-methoxyphenyl)-9H-pyrido[3,4-b]indole-3-carboxylate (7b). A pale yellow solid. 29%. ^{1}H NMR (400 MHz, d_6-DMSO) δ 3.88 (s, 3H), 3.92 (s, 3H), 7.12 – 7.15 (m, 1H), 7.32 (m, 1H), 7.57 – 7.61 (m, 1H), 7.52 (brs, 1H), 7.69 (d, J = 8.4 Hz, 1H), 8.42 (d, J = 8.0 Hz, 1H), 8.92 (s, 1H), 11.91 (s, 1H)

Methyl 1-(3-fluorophenyl)-9H-pyrido[3,4-b]indole-3-carboxylate (7c). A colourless solid. 53%. ^{1}H NMR (400 MHz, d_6-DMSO) δ 3.93 (s, 3H), 7.34 (dd, J = 7.6, 7.2 Hz, 1H), 7.40 (m, 1H), 7.59 – 7.65 (m, 1H), 7.69 (dd, J = 8.0, 6.4 Hz, 1H), 7.80 (d, J = 10 Hz, 1H), 7.87 (d, J = 7.2 Hz, 1H), 8.43 (d, J = 8.0 Hz, 1H), 8.95 (s, 1H), 11.98 (s, 1H)

Methyl 1-(3-chloropheny)-9H-pyrido[3,4-b]indole-3-carboxylate (7d). A colourless solid. 53%. ^{1}H NMR (300 MHz, d_6-DMSO) δ 3.93 (s, 3H), 7.33 (dd, J = 7.8, 6.9 Hz, 1H), 7.59 – 7.71 (m, 4H), 7.99 (dd, J = 8.7, 1.8 Hz, 1H), 8.01(s, 1H), 8.44 (d, J = 8.1 Hz, 1H), 8.95 (s, 1H), 12.04 (s, 1H). MS (ESI): calcd. for $C_{19}H_{14}ClN_2O_2$ $[M+H]^+$ 337.07; found 337.07

Methyl 1-(3-bromophenyl)-9H-pyrido[3,4-b]indole-3-carboxylate (7e). A pale yellow solid. 5%. ^{1}H NMR (400 MHz, d_6-DMSO) δ 3.93 (s, 3H), 7.34 (m, 1H), 7.60 (m, 2H), 7.63 (d, *J* = 3.2 Hz, 1H), 7.69 (d, *J* = 8.4 Hz, 1H), 7.77 (d, *J* = 9.2 Hz, 1H), 8.03 (d, *J* = 7.6 Hz, 1H), 8.13 (s, 1H), 8.44 (d, *J* = 7.6 Hz, 1H), 8.95 (s, 1H), 12.02 (s, 1H)

Methyl 1-(3-nitrophenyl)-9H-pyrido[3,4-b]indole-3-carboxylate (7f). A brownish yellow solid. 43%. ^{1}H NMR (400 MHz, d_6-DMSO) δ 3.94 (s, 3H), 7.35 (dd, *J* = 7.6, 7.2 Hz, 1H), 7.63 (dd, *J* = 8.0, 6.4 Hz, 1H), 7.70 (m, 1H), 7.93 (dd, *J* = 8.4, 8.0 Hz, 1H), 8.40 (d, *J* = 7.6 Hz, 1H), 8.46 (d, *J* = 7.6 Hz, 2H), 8.77 (s, 1H), 9.00 (s, 1H), 12.15 (s, 1H)

Methyl 1-(4-chlorophenyl)-9H-pyrido[3,4-b]indole-3-carboxylate (7g). A pale yellow solid. 9%. ^{1}H NMR (300 MHz, $CDCl_3$) δ 4.04 (s, 3H), 7.37 (dd, *J* = 7.8, 7.5 Hz, 1H), 7.55 (d, *J* = 8.4 Hz, 2H), 7.52 – 7.65 (m, 2H), 7.91 (d, *J* = 8.4 Hz, 2H), 8.17 (d, *J* = 7.5 Hz, 1H), 8.73 (s, 1H)

Methyl 1-(3,5-difluorophenyl)-9H-pyrido[3,4-b]indole-3-carboxylate (7h). A colourless solid. 67%. ^{1}H NMR (300 MHz, d_6-DMSO) δ 7.35 (t, *J* = 6.9 Hz, 1H), 7.46 (tt, *J* = 9.0, 2.1 Hz, 1H), 7.69 (brs, 2H), 7.60 – 7.71 (m, 2H), 8.45 (d, *J* = 8.1 Hz, 1H), 8.98 (s, 1H), 12.06 (s, 1H). HRMS (ESI): calcd. for $C_{19}H_{13}F_2N_2O_2$ $[M+H]^+$ 339.0945; found 339.0951.

Methyl 1-(3,5-dichlorophenyl)-9H-pyrido[3,4-b]indole-3-carboxylate (7i). A colourless solid. 53%. ^{1}H NMR (300 MHz, d_6-DMSO) δ 3.94 (3H, s), 7.35 (t, *J* = 7.8 Hz, 1H), 7.60 – 7.72 (m, 2H), 7.82 (t, *J* = 1.8 Hz, 1H), 7.98 (t, *J* = 1.8 Hz, 2H), 8.99 (s, 1H), 12.13 (s, 1H). HRMS (ESI): calcd. for $C_{19}H_{13}Cl_2N_2O_2$ $[M+H]^+$ 371.0354; found 371.0354

Methyl 1-(3,5-dibromophenyl)-9H-pyrido[3,4-b]indole-3-carboxylate (7j). A colourless solid. 70%. ^{1}H NMR (300 MHz, d_6-DMSO) δ 3.94 (s, 3H), 7.35 (t, *J* = 7.5 Hz, 1H), 7.60 – 7.72 (t, *J* = 7.5 Hz, 1H), 8.04 (brs, 1H), 8.13 (d, *J* = 1.8 Hz, 2H), 8.98(s, 2H), 8.45 (d, *J* = 7.8 Hz, 1H), 12.13 (s, 1H). HRMS (ESI): calcd. for $C_{19}H_{13}Br_2N_2O_2[M+H]^+$ 458.9344; found 458.9346

Synthesis of carboxamides 3, 4, 8a, b, e – n

General procedure. To a solution of carboxylate **7** (0.1 – 0.2 mmol) in MeOH–THF (1: 1, 10 – 20 ml) was added an excess amine (NH_4OH, NH_2OH, or alkylamine, 3 – 6 mmol), and the mixture was stirred for 2 – 3 days at room temperature or 45ºC. The reaction mixture was concentrated under reduced pressure, and a pale yellow or a brown residue was purified by silica gel column chromatography or PLC.

***N*-(3-Hydroxypropyl)-1-(3-chlorophenyl)-9H-pyrido[3,4-b]indole-3-carboxamide (3).** A colourless solid. 84%. ^{1}H NMR (400 MHz, $CDCl_3$) δ 1.66 (brs, 2H), 3.69 (brs, 4H), 7.34 (brs, 1H), 7.49 – 7.57 (m, 3H), 7.85 (m, 1H), 7.94 (brs, 1H), 8.15 (d, *J* = 8.4, 1H), 8.42 (brs, 1H), 8.84 (m, 1H), 8.92 (brs, 1H)

***N*-(2-Hydroxyethyl)-1-(3-chlorophenyl)-9H-pyrido[3,4-b]indole-3-carboxamide (4).** A colourless solid. 74%. ^{1}H NMR (300 MHz, $CDCl_3$) δ 3.70 (dt, *J* = 5.1, 5.1 Hz, 2H), 3.85 (t, *J* = 5.1 Hz, 2H), 7.40 – 7.60 (m, 1H), 7.90 (t, *J* = 1.8 Hz, 1H), 8.09 (d, *J* = 7.5 Hz, 1H), 8.10 (ddd, *J* = 7.5, 1.8, 1.2 Hz, 1H), 8.76 (s, 1H), 8.53 (brs, 1H), 8.84 (s, 1H)

1-(3-Chlorophenyl)-9H-pyrido[3,4-b]indole-3-carboxamide (8a)[1]. A pale yellow solid. 30%. ^{1}H NMR (300 MHz, d_6-DMSO) δ 7.32 (dd, *J* = 7.5, 7.2 Hz, 1H), 7.52 – 7.70 (m, 5H), 8.12 (d, *J* = 6.9 Hz, 1H), 8.21 (s, 2H), 8.40 (d, *J* = 7.5 Hz, 1H), 8.85 (s, 1H), 11.91 (s, 1H). MS (FAB): calcd. for $C_{18}H_{13}ClN_3O$ $[M+H]^+$ 322.07; found 322.03

***N*-Hydroxy-1-(3-chlorophenyl)-9H-pyrido[3,4-b]indole-3-carboxamide (8b).** A pale yellow solid. 18%. ^{1}H NMR (300 MHz, d_6-DMSO) δ 7.31 (t, *J* = 7.5 Hz, 1H), 7.57 – 7.70 (m, 4H), 8.16 (d, *J* = 7.5 Hz, 1H), 8.34 (brs, 1H), 8.42 (d, *J* = 7.5 Hz, 1H), 8.79 (s, 1H), 9.05 (s, 1H), 11.99(s, 1H). MS (FAB): calcd. for $C_{18}H_{12}ClN_3O_2$ $[M+H]^+$ 337.06; found 338.04

1-Phenyl-9H-pyrido[3,4-b]indole-3-carboxamide (8e). A colourless solid. 62%. ^{1}H NMR (500 MHz, d_6-DMSO) δ 7.31 (dd, *J* = 8.0, 8.0 Hz, 1H) 7.53 (brd, *J* = 3.0 Hz, 1H), 7.56 (tt, *J* = 7.5, 1.5 Hz, 1H), 7.59 (dd, *J* = 8.0, 1.0 Hz, 1H) 7.63 (dd, *J* = 7.5, 7.5 Hz, 2H), 7.68 (d, *J* = 8.0 Hz, 1H), 8.10 (brd, *J* = 3.0 Hz,1H), 8.16 (dd, *J* = 7.5, 1.5 Hz, 2H), 8.40 (d, *J* = 8.0 Hz, 1H), 8.83 (s, 1H), 11.83 (s, 1H). ^{13}C NMR (125 MHz, d_6-DMSO) δ 112.6, 113.1, 120.2, 121.2, 122.0, 128.6, 128.7, 128.8, 128.9, 129.9, 134.2, 137.5, 140.0, 140.5, 141.6, 167.0. HRMS (EI) calcd. for $C_{18}H_{13}N_3O$ $[M^+]$ 287.1059, found 287.1058

1-(3-Methoxyphenyl)-9H-pyrido[3,4-b]indole-3-carboxamide (8f). A pale yellow solid. 52%. ^{1}H NMR (400 MHz, d_6-DMSO) δ 7.12 (dd, *J* = 8.0, 2.4 Hz, 1H), 7.30 (dd, *J* = 7.6, 7.2 Hz, 1H), 11.80 (s, 1H), 7.51 (brs, 1H), 7.52 – 7.60 (m, 2H), 7.64 (brs, 1H), 7.68 (d, *J* = 8.8 Hz, 2H), 8.10 (s, 1H), 8.30 (s, 1H), 8.39 (d, *J* = 8.0 Hz, 1H), 8.83 (s, 1H). HRMS (EI): calcd. for $C_{19}H_{15}N_3O_2$ $[M^+]$ 317.1165; found 317.1165

1-(3-Fluorophenyl)-9H-pyrido[3,4-b]indole-3-carboxamide (8g). A colourless solid. 18%. ^{1}H NMR (400 MHz, d_6-DMSO) δ 7.32 (t, *J* = 7.6 Hz, 1H), 7.38 (m, 1H), 7.58 – 7.64 (m, 1H), 7.68 (dd, *J* = 8.8, 8.4 Hz, 1H), 8.01 (s, 1H), 8.02 (m, 1H), 8.18 (s, 1H), 8.40 (d, *J* = 8.0 Hz, 1H), 11.88 (s, 1H), 8.85 (s, 1H). HRMS (EI): calcd. for $C_{18}H_{12}FN_3O$ [M^+] 305.0964; found 305.0959

1-(3-Bromophenyl)-9H-pyrido[3,4-b]indole-3-carboxamide (8h)[1]. A brownish yellow solid. 31%. ^{1}H NMR (300 MHz, d_6-DMSO) δ 7.31 (dd, *J* = 8.1, 6.9 Hz, 1H), 7.51 – 7.62 (m, 3H), 7.69 – 7.75 (m, 2H), 8.12 – 8.20 (m, 2H), 8.32 (brs, 1H), 8.40 (d, *J* = 6.6 Hz, 2H), 8.85 (s, 1H), 11.90 (s, 1H). HRMS (EI): calcd. for $C_{18}H_{12}BrN_3O$ [M^+] 365.0160; found 365.0162

1-(3-Nitrophenyl)-9H-pyrido[3,4-b]indole-3-carboxamide (8i). A pale yellow solid. 26%. ^{1}H NMR (400 MHz, d_6-DMSO) δ 7.33 (t, *J* = 7.6 Hz, 1H), 7.56 (brs, 1H), 7.60 – 7.69 (m, 2H), 7.92 (dd, *J* = 8.4, 7.9 Hz, 1H), 8.21 (brs, 1H), 8.39 (d, *J* = 8.4 Hz, 1H), 8.44 (d, *J* = 7.2 Hz, 1H), 8.60 (d, *J* = 7.2 Hz, 1H), 8.87 (brs, 1H), 8.91 (s, 1H), 12.02 (s, 1H). HRMS (EI): calcd. for $C_{18}H_{12}N_4O_3$ [M^+] 332.0910; found 332.0906

1-(4-Chlorophenyl)-9H-pyrido[3,4-b]indole-3-carboxamide (8j)[1]. A pale yellow solid. 65%. ^{1}H NMR (300 MHz, CD_3OD) δ 7.24 (dd, *J* = 7.8, 6.9 Hz, 1H), 7.46 – 7.57 (m, 2H), 7.52 (d, *J* = 8.4 Hz, 2H), 7.99 (d, *J* = 8.4 Hz, 2H), 8.73 (d, *J* = 8.7 Hz, 1H). HRMS (ESI): calcd. for $C_{18}H_{13}ClN_3O$ $[M+H]^+$ 322.0747; found 322.0751

***N*-Hydroxy-(4-chlorophenyl)-9H-pyrido[3,4-b]indole-3-carboxamide (8k).** A pale yellow solid. 13%. ^{1}H NMR (300 MHz, CD_3OD) δ 7.33 (dd, *J* = 8.4, 7.5 Hz, 1H), 7.56 – 7.67 (m,

2H), 7.61 (d, *J* = 8.4 Hz, 2H), 7.83 (s, 1H), 8.09 (d, *J* = 8.4 Hz, 2H), 8.25 (d, *J* = 8.1 Hz, 1H), 8.77 (s, 1H). HRMS (ESI): calcd. for $C_{18}H_{13}ClN_3O$ $[M+H]^+$ 338.0696; found 338.0700

1-(3,5-Difluorophenyl)-9H-pyrido[3,4-b]indole-3-carboxamide (8l). A colourless solid. 38%. ^1H NMR (300 MHz, d_6-DMSO) δ 7.32 (t, *J* = 6,9 Hz, 1H), 7.42 (tt, *J* = 9.3, 2.1 Hz, 1H), 7.55 (brs, 1H), 7.59 – 7.72 (m, 2H), 7.94 (brd, *J* = 6.6 Hz, 2H), 8.28 (brs, 1H), 8.42 (d, *J* = 7.8 Hz, 1H), 8.88 (s, 1H), 11.97 (s, 1H). HRMS (ESI): calcd. for $C_{18}H_{12}F_2N_3O$ $[M+H]^+$ 324.0948; found 324.0955

1-(3,5-Dichlorophenyl)-9H-pyrido[3,4-b]indole-3-carboxamide (8m). A pale yellow solid. 29%. ^1H NMR (300 MHz, d_6-DMSO) δ 7.32 (t, *J* = 7.5 Hz, 1H), 7.53 (brs, 2H), 7.59 – 7.71 (m, 2H), 7.78 (t, *J* = 2.1 Hz, 1H), 8.18 (t, *J* = 2.1 Hz, 2H), 8.28 (brs, 1H), 8.42 (d, *J* = 7.8 Hz, 1H), 8.88 (s, 1H), 12.00 (s, 1H). HRMS (ESI): calcd. for $C_{18}H_{12}Cl_2N_3O$ $[M+H]^+$ 356.0357; found 356.0361

1-(3,5-Dibromophenyl)-9H-pyrido[3,4-b]indole-3-carboxamide (8n). A brown solid. 33%. ^1H NMR (300 MHz, d_6-DMSO) δ 7.32 (t, *J* = 7.8 Hz, 1H), 7.55 (brs, 2H), 7.59 – 7.71 (m, 2H), 8.01 (t, 1H, *J* = 1.8 Hz), 8.32 (d, *J* = 1.8 Hz, 2H), 8.41 (d, *J* = 8.1 Hz, 1H), 8.87 (s, 1H), 12.01 (s, 1H). HRMS (ESI): calcd. for $C_{18}H_{12}Br_2N_3O$ $[M+H]^+$ 443.9347; found 443.9343

Synthesis of acetate 8c. A solution of a hydroxypropyl derivative **3** (10 mg, 0.026 mmol) and Ac_2O (80 mg, 0.78 mmol) in dry pyridine (0.5 ml) was stirred overnight at room temperature under N_2. Water was added and the mixture was stirred vigorously for 5 min. The aqueous solution was extracted 3 times with dichloromethane. The combined organic layers were dried over Na_2SO_4 and evaporated to give crystalline solid, which was further purified by PLC (hexane: ethyl acetate = 1: 1) to afford the acetate **8c** (10 mg, 90%) as a

colourless solid. ^{1}H NMR (300 MHz, $CDCl_3$) δ 1.23 (m, 2H), 2.00 (s, 3H), 3.63 (dt, *J* = 6.6, 6.6 Hz, 2H), 4.20 (t, *J* = 6.6 Hz, 2H), 7.35 (dd, *J* = 8.1, 6.3 Hz, 1H), 7.47 – 7.61 (m, 4H), 7.85 (d, *J* = 7.5 Hz, 1H), 7.95 (s, 1H), 8.19 (d, *J* = 7.8 Hz, 1H), 8.36 (brs, 1H), 8.76 (s, 1H), 8.88 (s, 1H). MS (FAB): calcd. for $C_{23}H_{21}ClN_3O_3$ $[M+H]^+$ 422.12; found 422.10

Synthesis of methyl ether 8d. To a stirred solution of a hydroxypropyl derivative **3** (10 mg, 0.026 mmol) in CH_2Cl_2 (2 ml) was added 48% aqueous fluoroboric acid (10 µl, 0.053 mmol) and trimethylsilyldiazomethane[2] (0.6 mol/l in hexane; 0.1 ml, 0.06 mmol) at 0ºC. Water was added after stirring for 30 min. The whole mixture was stirred vigorously for 30 min and neutralized with a 0.1 M $NaHCO_3$ aqueous solution. The mixture was extracted 3 times with CH_2Cl_2. The combined organic layers were dried over Na_2SO_4 and evaporated to give a crystalline solid, which was further purified by PLC (hexane: ethyl acetate = 1: 1) to afford the methyl ether **8d** (3 mg, 29%) as a colourless solid. ^{1}H NMR (300 MHz, $CDCl_3$) δ 1.90 – 1.98 (m, 2H), 3.38 (s, 3H), 3.56 (t, *J* = 6.3 Hz, 2H), 3.64 (dt, *J* = 6.3, 6.3 Hz, 2H), 7.35 (ddd, *J* = 8.1, 6.6, 1.5 Hz, 1H), 7.47 – 7.61 (m, 4H), 7.87 (d, *J* = 7.8 Hz, 1H), 8.01 (s, 1H), 8.20 (d, *J* = 7.8 Hz, 1H), 8.59 (brs, 1H), 8.69 (s, 1H), 8.90 (s, 1H). MS (FAB): calcd. for $C_{22}H_{21}ClN_3O_2$ $[M+H]^+$ 394.12; found 394.10

Synthesis of carboxylic acid 8r. To a stirred suspension of **7a** (286 mg, 0.95 mmol) in MeOH (30 ml) was added 1 N LiOH aqueous solution (4.7 ml, 4.7 mmol). The mixture was heated under reflux for 1 h. After cooling, the MeOH was evaporated. The residue was diluted with H_2O, acidified with 1 N HCl, and extracted with ethyl acetate. The combined organic layers were washed with brine, dried over Na_2SO_4, and evaporated under reduced pressure to afford the carboxylic acid **8r** (260 mg, 95%) as a pale yellow solid. ^{1}H NMR (500 MHz, CD_3OD) δ 7.38 (ddd, *J* = 8.0, 8.0, 1.1 Hz, 1H), 7.59 (tt, *J* = 7.4, 2.3 Hz, 1H), 7.63 (ddd,

J = 8.0, 8.0, 1.1 Hz, 1H), 7.65 (ddd, *J* = 7.4, 7.4, 1.1 Hz, 2H), 7.68 (bd, *J* = 8.0 Hz, 1H), 8.03 (ddd, *J* = 7.4, 1.7, 1.1 Hz, 2H), 8.33 (dd, *J* = 8.0, 1.1 Hz, 1H), 8.94 (s, 1H)

Synthesis of carboxylates 10a – c. Carboxylates **10a** – **c** were prepared according to the general procedure for **7a** – **j**.

Methyl 1-cyclohexyl-9-H-pyrido[3,4-b]indole-3-carboxylate (10a). A colourless solid. 51%, ^{1}H NMR (400 MHz, d_6-DMSO) δ 1.30 – 1.56 (m, 2H), 1.73 – 1.93 (m, 8H), 3.28 – 3.37 (m, 1H), 3.89 (s, 3H), 7.28 (dd, *J* = 7.9, 7.1 Hz, 1H), 7.57 (dd, *J* = 7.9, 7.1 Hz, 1H), 7.65(d, *J* = 8.3 Hz, 1H), 8.33(d, *J* = 7.9 Hz, 1H), 8.74 (s, 1H), 11.97 (s, 1H). HRMS (ESI): calcd. for $C_{19}H_{21}N_2O_2$ $[M+H]^+$ 309.1603; found 309.1597

Methyl 1-(furan-2-yl)-9-H-pyrido[3,4-b]indole-3-carboxylate (10b). A colourless solid, 41%. ^{1}H NMR (300 MHz, d_6-DMSO) δ 4.04 (s, 3H), 6.67 (dd, *J* = 4.0, 2.0 Hz, 1H), 7.36 (ddd, *J* = 8.3, 7.9, 1.9 Hz, 1H), 7.43 (d, *J* = 4.0 Hz, 1H), 7.60 (dd, *J* = 8.3, 7.1 Hz, 1H), 7.61 (d, *J* = 7.1Hz, 1H), 7.72 (d, *J* = 0.8 Hz, 1H), 8.18 (d, *J* = 7.9 Hz, 1H), 8.79 (s, 1H), 9.58 (s, 1H). HRMS (ESI): calcd. for $C_{17}H_{13}N_2O_3$ $[M+H]^+$ 293.0926; found 293.0932

Methyl 1-(pyridine-4-yl)-9-H-pyrido[3,4-b]indole-3-carboxylate (10c). A colourless solid. 67%. ^{1}H NMR (400 MHz, d_6-DMSO) δ 3.93 (s, 3H), 7.35 (brt, *J* = 8.3 Hz, 1H), 7.63 (brt, *J* = 8.3 Hz, 1H), 7.70 (brd, *J* = 8.3 Hz, 1H), 8.00 (d, *J* = 4.3 Hz, 2H), 8.46 (d, 1 *J* = 8.3 Hz, 1H), 8.83 (d, *J* = 4.3 Hz, 2H), 9.07 (s, 1H), 12.08 (s, 1H). HRMS (ESI): calcd. for $C_{18}H_{14}N_3O_2$ $[M+H]^+$ 304.1086; found 304.1081

Synthesis of carboxamides 11a – c. Carboxamides **11a** – **c** were prepared according to the general procedure for **3**, **4**, and **8a**, **b**, **e** – **n**.

1-Cyclohexyl-9H-pyrido[3,4-b]indole-3-carboxamide (11a). A colourless solid, 45%. ^{1}H NMR (300 MHz, d_6-DMSO) δ 1.32 – 1.60 (m, 2H), 1.74 – 1.98 (m, 8H), 3.27 – 3.38 (m, 1H), 7.26 (dd, *J* = 7.8, 7.2 Hz, 1H), 7.44 (brd, *J* = 3.3 Hz, 1H), 7.55 (dd, *J* = 7.8, 7.2 Hz, 1H), 7.63 (d, 1H, *J* = 8.1Hz), 7.95 (brd, *J* = 3.3 Hz, 1H), 8.31 (d, *J* = 7.5 Hz, 1H), 8.65 (s, 1H), 11.88 (s, 1H). HRMS (ESI): calcd. for $C_{18}H_{20}N_3O$ $[M+H]^+$ 294.1606; found 294.1600

1-(Furan-2-yl)-9H-pyrido[3,4-b]indole-3-carboxamide (11b)[2]. A colourless powder, 87%. ^{1}H NMR (400 MHz, d_6-DMSO) δ 6.82 (brs, 1H), 7.30 (brt, *J* = 7.9 Hz, 1H), 7.54 (brs, 1H), 7.60 (brt, *J* = 7.5 Hz, 1H), 7.66 (brs, 1H), 7.79 (d, *J* = 7.9 Hz, 1H), 8.01 (brs, 1H), 8.25 (brs, 1H), 8.37 (d, *J* = 7.5 Hz, 1H), 8.76 (s, 1H), 11.77 (s, 2H). HRMS (ESI): calculated for $C_{16}H_{12}N_3O_2$ $[M+H]^+$ 278.0930; found 278.0937

1-(Pyridine-4-yl)-9H-pyrido[3,4-b]indole-3-carboxamide (11c). A colourless solid. 68%. ^{1}H NMR (400 MHz, d_6-DMSO) δ 7.33 (dd, *J* = 7.9, 7.1 Hz, 1H), 7.58 (brs, 1H), 7.62 (dd, *J* = 8.3, 7.1 Hz, 1H), 7.69 (d, *J* = 8.3 Hz, 1H), 8.11 (d, *J* = 5.1 Hz, 1H), 8.21 (brs, 1H), 8.43 (d, *J* = 7.9 Hz, 1H), 8.82 (d, *J* = 5.1 Hz, 2H), 8.92 (s, 1H), 11.99 (s, 1H). HRMS (ESI): calcd. for $C_{17}H_{12}N_4ONa$ $[M+Na]^+$ 311.0909; found 311.0902

Synthesis of tetahydro-β-carbolines 12a – f

***(S)*-2,3,4,9-tetrahydro-1H-pyrido[3,4-b]indole-3-carboxylic acid (12a)[3].** To a solution of L-tryptophan (204.4 mg, 1 mmol) in NaOH solution (0.4N, 5 ml) 37% HCHO solution (80 µl, 1 mmol) was added at 0ºC, and the whole mixture was stirred overnight at room temperature.

The resulting white solid was collected after acidification (pH ~4) with acetic acid (AcOH) and washed well with distilled water to give 190 mg (87%). ^{1}H NMR (300 MHz, D_6-DMSO) δ 2.80 (dd, *J* = 15.6, 10.8 Hz, 1H), 3.12 (dd, *J* = 15.6, 5.4 Hz), 3.59 (dd, *J* = 10.8, 5.4 Hz, 1H), 4.15 (d, *J* = 14.4, 1H), 4.22 (d, *J* = 14.4, 1H), 6.97 (dd, *J* = 7.8, 6.9 Hz, 1H), 7.06 (dd, *J* = 8.1, 6.9 Hz, 1H), 7.31 (d, *J* = 8.1 Hz, 1H), 7.43 (d, *J* = 7.8 Hz, 1H)

(*S*)-2,3,4,9-tetrahydro-1H-pyrido[3,4-b]indole-3-carboxamide (12b)[4] & (*S*)-9-(hydroxymethyl)- 2,3,4,9-tetrahydro-1H-pyrido[3,4-b]indole-3-carboxamide (12d). To a solution of tryptophanamide hydrochloride (119.8 mg, 0.5 mmol) in MeOH (1 ml) was added 0.4 N NaOH solution (1.25 ml, 0.5 mmol) and 37% HCHO solution (40 μL, 0.5 mmol). The whole mixture was stirred overnight at room temperature. Subsequently, the solvent was evaporated to dryness and charged to a column of NH-silica gel (10 g). Column chromatography (CH_3Cl: MeOH = 5: 0.1 – 0.3) gave (**12b**, 55 mg, 51%) and (**12d**, 11 mg, 8.9%) as a colourless solid.

^{1}H NMR spectrum of **(12b)**: (300 MHz, d_6-DMSO) δ 2.61 (dd, *J* = 15.0, 11.9 Hz, 1H), 2.86 (dd, *J* = 15.0, 4.3 Hz, 1H), 3.38 (dd, *J* = 11.9, 4.3 Hz, 1H), 3.88 (d, 1H, *J* = 16.6 Hz, 1H), 3.94 (d, *J* = 16.6 Hz, 1H), 6.91 (brt, *J* = 7.9 Hz, 1H). 6.98 (brt, *J* = 7.5 Hz, 1H), 7.25 (d, *J* = 7.5 Hz, 1H), 7.35 (d, *J* = 7.9 Hz, 1H), 10.66 (s, 1H)

^{1}H NMR spectrum of **(12d)**: (300 MHz, d_6-DMSO) δ 2.64 (dd, *J* = 15.0, 9.0 Hz, 1H), 2.88 (dd, *J* = 15.0, 4.5 Hz, 1H), 3.39 (dd, *J* = 9.0, 4.5 Hz, 1H), 3.92 (d, 1H, *J* = 16.8 Hz, 1H), 4.08 (d, *J* = 16.8 Hz, 1H), 5.38 (s, 2H), 7.02 (brt, *J* = 7.5 Hz, 1H). 7.10 (brt, *J* = 8.1 Hz, 1H), 7.41 (d, *J* = 7.8 Hz, 1H), 7.50 (d, *J* = 8.1 Hz, 1H)

(*S*)-9-(hydroxymethyl)- 2,3,4,9-tetrahydro-1H-pyrido[3,4-b]indole-3-carboxylic acid (12c). To a solution of L-tryptophan (2 g, 9.8 mmol) in 0.4 N NaOH solution (24.5 ml, 9.8 mmol) was added 37% HCHO solution (1.12 ml, 13.8 mmol, 1.4 equivalents). The whole mixture was stirred overnight at room temperature. After further stirring for 1 day, the reaction mixture was acidified with AcOH (0.8 ml) to pH 4 – 5 to give a white precipitate, which was collected and washed well with cold water and dried. ^{1}H NMR (400 MHz, d_6-DMSO) δ 2.83 (dd, *J* = 15.4, 9.9 Hz, 1H), 3.11 (dd, *J* = 15.4, 5.1 Hz, 1H), 3.58 (dd, *J* = 9.9, 5.1 Hz, 1H), 4.26 (d, *J* = 16.2 Hz, 1H), 4.40 (d, *J* = 16.2 Hz, 1H), 5.41 (s, 2H), 7.04 (brt, *J* = 7.1 Hz, 1H), 7.14 (brt, *J* = 6.7 Hz, 1H), 7.46 (d, *J* = 7.5 Hz, 1H). ^{13}C NMR (d_6-DMSO) δ 22.8, 56.3, 65.5, 107.5, 109.9, 117.9, 119.4, 121.5, 126.3, 128.7, 136.4, 169.7

(3S)-1-phenyl-2,3,4,9-tetrahydro-1H-pyrido[3,4-b]indole-3-carboxamide (12e). To a solution of L-tryptophan methyl ester (218 mg) in an anhydrous methylene chloride, CF_3COOH (60 μL) and benzaldehyde (130 mg) was added and stirred overnight at room temperature. The whole mixture was evaporated to dryness, and purification of the residue with an NH-silica gel column (hexane: ethyl acetate = 1: 1) gave an intermediate adduct during the synthesis of **7a** mentioned above. The methyl ester obtained (59 mg) was treated with concentrated NH_4OH solution (2 ml) overnight at room temperature. Subsequently, the whole mixture was evaporated to dryness and purified over an NH-silica gel column ($CHCl_3$: MeOH = 5: 0.3), yielding carboxamide **12e** (43 mg, 77%) as colourless solid. ^{1}H NMR (400 MHz, $CDCl_3$) δ 2.94 (dd, *J* = 15.8, 9.1 Hz, 1H), 3.28 (dd, *J* = 15.8, 4.7 Hz, 1H), 3.65 (dd, *J* = 9.1, 4.7 Hz, 1H), 5.25 & 5.28 (brs, 1H), 5.50 (brs, 1H), 6.80 (brs, 1H), 7.1 – 7.4 (m, 8H), 7.56 (d, *J* = 7.5 Hz, 1H), 7.76 (brs, 1H)

(*S*)-1-(1,2-dihydroxyethyl)-2,3,4,9-tetrahydoro-1H-pyrido[3,4-b]indole-3-carboxylic acid (12f). To a solution of L-tryptophan (102.2 mg, 0.5 mmol) in 0.1 N H_2SO_4 solution (2 ml)

was added DL-glyceraldehyde (54 mg, 0.6 mmol). The reaction mixture was monitored in an SiO_2 TLC plate with butanol: acetic acid: water solution (4: 2: 1 ratio) and stirred overnight at room temperature. The resulting white crystalline solid was collected by filtration and washed well with cold water to afford 89.5 mg of a crude mixture, which was further purified over a Cosmosil column with H_2O: MeOH (30: 3 – 12). Lyophilisation of the fraction gave (**12f**) as an amorphous powder (44 mg, 32%). ^1H NMR (400 MHz, d_6-DMSO) δ 2.6 – 2.8 (m, 2.5H), 3.4 – 4.1 (m, 3.5H), 4.38 (d, J = 11.9 Hz, 0.6H), 4.5 (d, J = 3.9 Hz, 0.4H), 6.94 (brt, J = 6.7 Hz, 0.4H), 6.95 (brt, J = 6.3 Hz, 0.6H), 7.02 (brt, J = 5.9 Hz, 0.6H), 7.04 (brt, J = 7.1 Hz, 0.4H), 7.33 (d, J = 7.5 Hz, 0.6H), 7.34 (d, J = 4.7 Hz, 0.4H), 7.37 (d, J = 4.0 Hz, 0.4H), 7.41 (d, J = 7.9 Hz, 0.6H). HRMS (ESI): calcd. for $C_{14}H_{17}N_2O_4$ $[M + H]^+$ 277.1188; found 277.1182

Supporting References

1. Srivastava, S. K.; Agarwal, A.; Chauhan, P. M.; Agarwal, S. K.; Bhaduri, A. P.; Singh, S. N.; Fatima, N.; Chatterjee, R. K., Potent 1,3-disubstituted-9H-pyrido[3,4-b]indoles as new lead compounds in antifilarial chemotherapy. *J. Med. Chem.* **1999,** *42* (6), 1667–1672.
2. Tang, J.-G.; Wang, Y.-H.; Wang, R.-R.; Dong, Z.-J.; Yang, L.-M.; Zheng, Y.-T.; Liu, J.-K., Synthesis of analogues of flazin, in particular, flazinamide, as promising anti-HIV agents. *Chem. Biodivers.* **2008,** *5* (3), 447-460.
3. Arnold, B.; Antonino, F.; Sidney, T., Alkaloids in mammalian tissues. 3. Condensation of L-tryptophan and L-5-hydroxytryptophan with formaldehyde and acetaldehyde. *J. Med. Chem.* **1973**, 418–420.
4. Saiga, Y.; Iijima, I.; Ishida, A.; Miyagishima, T.; Homma, K.; Oh-ishi, T.; Matsumoto, M.; Matsuoka, Y., Synthesis of 1,2,3,4-tetrahydro-beta-carboline derivatives as hepatoprotective agents. III.

Introduction of substituents onto methyl 1,2,3,4-tetrahydro-beta-carboline-2-carbodithioate.

Chem. Pharm. Bull. **1987,** *35*, 3284–3291.
